# Taking the Scenic Route: Polyomaviruses Utilize Multiple Pathways to Reach the Same Destination

**DOI:** 10.3390/v12101168

**Published:** 2020-10-15

**Authors:** Colleen L. Mayberry, Melissa S. Maginnis

**Affiliations:** 1 Department of Molecular and Biomedical Sciences, The University of Maine, Orono, ME 04469, USA; colleen.mayberry@maine.edu; 2 Graduate School in Biomedical Sciences and Engineering, The University of Maine, Orono, ME 04469, USA

**Keywords:** polyomavirus, viral entry, viral trafficking, viral disassembly, JC polyomavirus, BK polyomavirus, Merkel cell polyomavirus, mouse polyomavirus, SV40 polyomavirus, sialic acid receptors, clathrin-mediated endocytosis, non-clathrin, non-caveolae mediated endocytosis, ER, retrograde transport

## Abstract

Members of the *Polyomaviridae* family differ in their host range, pathogenesis, and disease severity. To date, some of the most studied polyomaviruses include human JC, BK, and Merkel cell polyomavirus and non-human subspecies murine and simian virus 40 (SV40) polyomavirus. Although dichotomies in host range and pathogenesis exist, overlapping features of the infectious cycle illuminate the similarities within this virus family. Of particular interest to human health, JC, BK, and Merkel cell polyomavirus have all been linked to critical, often fatal, illnesses, emphasizing the importance of understanding the underlying viral infections that result in the onset of these diseases. As there are significant overlaps in the capacity of polyomaviruses to cause disease in their respective hosts, recent advancements in characterizing the infectious life cycle of non-human murine and SV40 polyomaviruses are key to understanding diseases caused by their human counterparts. This review focuses on the molecular mechanisms by which different polyomaviruses hijack cellular processes to attach to host cells, internalize, traffic within the cytoplasm, and disassemble within the endoplasmic reticulum (ER), prior to delivery to the nucleus for viral replication. Unraveling the fundamental processes that facilitate polyomavirus infection provides deeper insight into the conserved mechanisms of the infectious process shared within this virus family, while also highlighting critical unique viral features.

## 1. Introduction

In order to infect a host, all viruses must transverse cellular barriers to access the interior of the cell where viruses replicate and propagate infection [1,2]. While these barriers exist, viruses have developed unique mechanisms to enter into a host cell and traffic within the cytoplasm to reach the appropriate compartment for replication [1,2]. Despite this, not all virions are capable of productive infection. During nonproductive infection, the virus may be shuttled to the incorrect compartment or targeted for degradation. Most often, this compartment does not support the progression of infection [1]. Furthermore, nonproductive routes of infection may result in identification of the virus by the host immune system, resulting in targeting of the virus and clearance of the infection. To avoid this fate, viruses have developed mechanisms to orchestrate attachment, entry, and trafficking to the appropriate compartment that supports either fusion or penetration for release of the viral genetic material.

*Polyomaviridae* comprises a family of 14 human polyomaviruses [3] that are classified according to their morphological and genetic similarities. Polyomaviruses are dsDNA viruses containing genomes of roughly 5000 base pairs. Their virions are approximately 40–45 nm in diameter with an icosahedral structure and lack a viral envelope [4,5,6]. The capsids of these viruses are comprised primarily of viral protein 1 (VP1), and secondarily of VP2 and VP3 [4,7]. Due to their importance in human health, infection strategies of polyomaviruses BK (BKPyV), JC (JCPyV), and Merkel cell (MCPyV) are of significant interest. However, advances in this field can be largely attributed to studies on nonhuman polyomaviruses, murine (mPyV) and SV40 [8]. While not discussed in detail in this review, other human polyomaviruses have been implicated with human disease including TSPyV, associated with the disease *Trichodysplasia spinulosa*, a rare skin condition [9]. Furthermore, human polyomaviruses 6, 7, and 9 (HPyV6, HPyV7, HPyV9), originally isolated from the skin, have not been associated with the causation of any human pathology [10], though DNA from these polyomaviruses have been detected in cutaneous T-cell lymphomas [11]. Additionally, New Jersey polyomavirus (NJPyV), though unconfirmed, may be associated with vasiculitic myopathy, a condition affecting skeletal muscle [12]. BKPyV establishes an asymptomatic infection in the renourinary tract of healthy individuals. In spite of this, under conditions of immunosuppression, BKPyV may cause nephropathy and hemorrhagic cystitis; individuals at particular risk for BKPyV-associated disease are those who have received an organ transplant [13]. In comparison, JCPyV also establishes a lifelong infection within the kidney of healthy individuals [6]. In immunosuppressed individuals, JCPyV can cause progressive multifocal leukoencephalopathy (PML), a fatal demyelinating disease affecting the central nervous system (CNS) [6]. MCPyV, discovered in 2008, can cause Merkel Cell carcinoma, an aggressive, albeit rare, form of skin cancer [14]. While there are significant commonalities between these viruses, they differ in their utilization of cellular receptors and entry routes for initiating infection. However, one distinct overlap in infection is clear: all polyomaviruses studied to date traffic to the endoplasmic reticulum following attachment and entry [15,16,17,18,19,20,21].

This review details the mechanisms by which several human and non-human polyomaviruses attach and transverse biologically relevant membranes, traffic through the cytoplasm to the endoplasmic reticulum, and uncoat for delivery of the virion to the nucleus for viral replication. Understanding the mechanisms by which polyomaviruses arrive at the nucleus could identify targets for the prevention of infection and spread of disease.

## 2. Attachment

**Figure 1:**
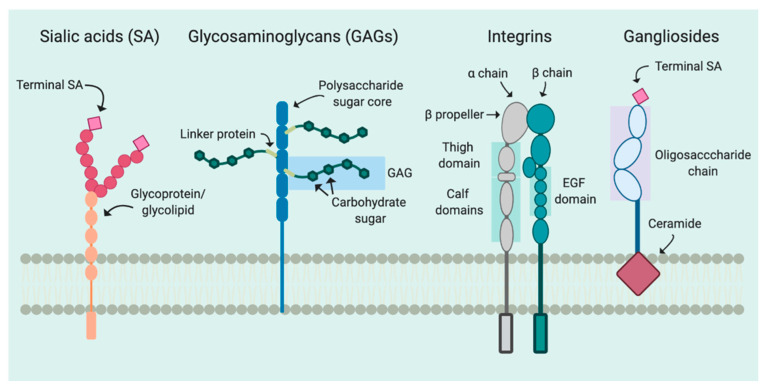
Attachment factors commonly used by polyomaviruses. Sialic acid-containing receptors typically consist of glycoproteins or glycolipids (orange) with attached mono or oligosaccharide backbones (pink circles) and terminal sialic acid residues (pink diamonds). Glycosaminoglycans contain repeating disaccharides (green hexagons) and are attached to the polysaccharide sugar core (blue) by link proteins (light green). Integrins are heterodimeric proteins comprised of α and β chains containing β domains attached to thigh and calf domain regions. β chains further contain epidermal growth factor (EGF) binding domains. Gangliosides are glycosphingolipids with a ceramide moiety embedded in the lipid bilayer and an oligosaccharide chain containing one or more sialic acids. Gangliosides may also contain a terminal sialic acid residue (pink diamond). Figure created with BioRender.com.

Most often, the initiation of viral infection is mediated by interactions between viruses and specific cellular factors facilitating viral attachment [1]. Attachment can directly induce structural conformational changes or activate specific signaling pathways to allow for uptake of the virus within the cell [22,23]. Because of this, more than one receptor may be necessary to allow for coordination of virus entry. Furthermore, interactions with cellular receptors largely dictate the signaling networks critical for delivery of the virus to the appropriate cellular compartment, ensuring replicative success [5]. Many viruses utilize attachment factors such as sialic acid-containing receptors including glycoproteins or glycolipids, or glycosaminoglycans and integrins (Figure 1), and this conserved attachment strategy extends to polyomaviruses [1,5].

The cell surface is decorated with glycoconjugates, carbohydrates covalently attached to biological molecules in the cell membrane, such as proteins or lipids [24]. The glycan chain of glycoproteins and glycolipids is often terminated by a sialic acid [25]. For example, glycolipids are composed of mono or oligosaccharides attached to a lipid moiety embedded in lipid-rich areas of the cell membrane [26]. Common glycolipids include the glycosphingolipids, gangliosides, which are comprised of oligosaccharides with one or more sialic acids attached to a ceramide moiety anchored in the plasma membrane [27]. Gangliosides are a large group of glycosphingolipids, referred to as a, b, or c series gangliosides, based on their sialic acid branching patterns [28]. Sialic acids are neuraminic acid derivatives with either an N- or and O-substitution and are comprised of a 9-carbon monosaccharide backbone [29]. Furthermore, sialic acids may be additionally modified on their hydroxyl group through acetylation or methylation [30,31]. The most common linkages are through either an α2,3 or α2,6 at the 2-carbon position of the internal Gal or GalNAc [29]. Glycosaminoglycans (GAGs) are long polysaccharide chains with a repeating disaccharide containing an amino sugar [32]. Integrins are comprised of heterodimers of α and β subunits. Though they primarily function as adhesion molecules in cell–cell interactions, integrins are recognized as attachment and entry factors for many viruses [5,33]. Interactions between viruses and host cells are initiated through the aforementioned receptors; their distribution and diversity can have significant implications in tissue tropism and host range [34]. A deeper understanding of the attachment factors critical for replicative success for polyomaviruses will give increased insight into a virus family that, in spite of having a narrow host range and tissue tropism, can cause a wide range of disease pathologies.

Interestingly, all polyomaviruses studied to date use sialic acid-containing receptors for attachment to the host cell and/or entry [35,36]. This binding has been demonstrated to occur with at least one of the three known sialic acid linkages, α2,3, α2,6, or α2,8, with both mPyV and SV40 binding to α2,3-sialic acid containing receptors [37,38,39,40,41,42,43,44,45,46,47,48]. mPyV has more recently been identified to also bind to an α2,8-sialic acid containing receptor [49]. These initial interactions between the virus and host cell receptors occur through VP1, the major capsid component and the only protein expressed on the exterior surface [4,50]. Visualization of mPyV VP1 in complex with receptor portions was instrumental in understanding the interactions between the viral capsid and cellular sugar residues [39,51]. VP1 proteins are comprised of two anti-parallel β sheets [39,51]; the residues of the capsid implicated in binding with either α2,3- or α2,6-sialic acids were identified through at least three direct pocket interactions with VP1 [37,38,39,51]. Mutagenesis of the internal residues of the binding pockets results in loss of infectivity of mPyV [52,53]. Some of the cellular receptors reviewed in this work were identified through the implementation of binding and flotation assays, techniques that investigate the specific protein–protein interactions between viruses and sections of cellular membranes through fractionation [54]. Utilizing these techniques, interactions between mPyV and gangliosides GT1a, GD1a, or GT1b containing a terminal sialic acid residue were determined [55,56,57]. Each of these receptors was further characterized for their importance in mPyV infectivity in mouse embryo fibroblasts, as well as the identification of an additional α2,8-sialic acid containing receptor [49]. Membrane flotation and binding assays to identify protein-protein interactions were also used to demonstrate that ganglioside GM1 containing an α2,3-sialic acid linkage is the cellular attachment factor for SV40 [56]. Alternative studies suggest that MHC I may also play a role in cellular attachment and/or entry [58,59,60,61]. Several studies have challenged the role of MHC I in promoting SV40 attachment and/or entry [47,62], further supported by the demonstration that MHC I does not enter with SV40 [60]. In addition, the importance of GM1 as the primary receptor for SV40 was emphasized through the utilization of high-resolution crystallization [63]. Furthermore, association of SV40 with GM1 is sufficient to induce membrane curvature resulting in the formation of deep invaginations at the cell surface [64].

Curiously, α4 integrins have also been identified as attachment factors for mPyV. While typically responsible for regulating cellular attachment to the extracellular matrix, cytoskeletal rearrangements, and proliferation, knockdown of α4 integrins significantly reduces mPyV infection [52,65]. Furthermore, VP1 of mPyV contains an α4 integrin-binding motif, and mutagenesis of this motif reduces viral infectivity [66]. Binding to both gangliosides and α4 integrins is critical for activation of phosphoinositide-3 kinase/mitogen-activated protein kinase (PI3K/MAPK) signaling networks, both necessary for viral infection; yet, α4 integrins may not come into play until after interaction with gangliosides has occurred [67].

BKPyV requires interactions with sialic acid residues for productive infection. Initial investigation into the cellular factors necessary for BKPyV attachment was employed through the use of sialidase S and neuraminidase, which remove α2,3 or α2,3 and α2,6 sialic acids, respectively, resulting in the inhibition of infection in Vero cells [68]. Additionally, binding assays using ganglioside-containing liposomes revealed that GD1b and GT1b support viral attachment through interactions between terminal α2,8-disialic acid motifs and VP1 [48]. Through a structure–function analysis, BKPyV was demonstrated to require the α2,8-disialic acid motifs for binding, and b-series gangliosides GD2, GD3, GD1b, and GT1b, containing this epitope can support infection. X-ray crystal structure analysis of BKPyV VP1 in complex with GD3 revealed that Lys68 makes specific contacts with two sialic acid moieties of GD3 [69]. The binding capacities of different strains of BKPyV may have altered attachment properties and virulence in vivo due to alterations in amino acid sequences in the binding pockets of VP1 [70]. Interestingly, through mutagenesis of a single residue in VP1 of BKPyV, Lys68, attachment preference can be switched from ganglioside GD3 to GM1, the primary attachment receptor for SV40. Likewise, mutagenesis of the Ser68 residue of SV40 can switch attachment receptor preference from GM1 to GD3, the primary attachment receptor for BKPyV [63,69]. The capability of retargeting attachment and receptor switching of either SV40 or BKPyV through mutagenesis of a single capsid residue suggests a conservation of conformation and structure among VP1 capsid proteins among polyomaviruses, regardless of host range.

Cellular attachment of MCPyV relies on N-sulfated- or 6-O-sulfated heparin sulfate-containing GAGs in addition to ganglioside GT1b [71,72]. Furthermore, interaction with sialylated glycans occurs primarily between capsid protein VP1 and disaccharide Neu5Ac-α2,3-Gal in a conformation that differs from other polyomaviruses in complex with sialic acid-containing receptors [40]. Direct interaction with sialic acids does not interfere with MCPyV engagement of GAGs, suggesting that this complex occurs prior to interaction with sialic acids in a co-receptor attachment model [40].

JCPyV has been demonstrated to bind to GAGs [73] and either α2,3- or α2,6-terminal sialic acid receptors [41,42,43,44,45,46]. JCPyV was shown to utilize sialic-acid-containing gangliosides [43,46], yet the virus does not bind to gangliosides with high affinity [74], instead preferring the α2,6-sialic acid-containing lactoseries tetrasaccharide c (LSTc) on glycoproteins or glycolipids [45]. Furthermore, VP1 of JCPyV undergoes structural modifications for enhanced binding to LSTc, demonstrating further specificity for this receptor motif [45]. Interestingly, VP1 proteins of SV40, BKPyV, and JCPyV have a greater than 74% sequence identity and engage their sialic acid-containing attachment factors through highly conserved interactions between the VP1 and the terminal sialic acid residue of their respective receptors [35,63,69]. Importantly, the interactions between these viruses and their unique receptors is dependent on interactions between VP1 and the terminally expressed Neu5Ac outside of the sialic acid binding pocket, conferring specificity for these receptors in attachment and thus, productive infection [45,63,75].

Cellular attachment of other human polyomaviruses has also been elucidated. Interestingly, HPyV6 and HPyV7, common viral inhabitants of human skin, have elongated loops in their VP1 protein that obstruct sialic acid binding sites commonly utilized by other polyomaviruses [76]. Because of this blockade, HPyV6 and HPyV7 engage nonsialylated glycans for cellular attachment [76]. However, HPyV12 and TSPyV both engage α2,3- and α2,6-siaylated glycans, while NJPyV binds sialylated glyans in a previously known sialic acid binding site on VP1 [77]. Further, B-lymphotrophic polyomavirus (LPyV), a simian polyomavirus, which has been isolated from both monkey and human cells [78], also engages sialylated glycans for cellular attachment [79]. Largely, aside from these viruses utilizing glycans for cellular attachment, mechanisms associated with their cellular entry downstream of attachment remain mostly uncharacterized. Collectively, six distinct binding conformations between VP1 of the aforementioned polyomaviruses and their respective sialic acid-containing cellular receptors have been classified according to their architectural similarities accounting not only for the location of interaction on the VP1 protein, but also for the conformation of the sialic-acid within the binding pocket: (1) for BKPyV, SV40, and JCPyV, (2) for mPyV, (3) for MCPyV, (4) for LPyV and HPyV9, (5) for TSPyV, and lastly, the most recently identified conformation, (6) for NJPyV [77]. Therefore, while human viruses within this family engage distinct receptors for attachment, they do so with a high degree of conservation and specificity.

## 3. Entry

Host cell factors may serve as either virus attachment moieties or functional receptors that can coordinate internalization of the virus within the cell. However, in some instances, interactions between viruses and primary receptors are not sufficient to activate the internalization machinery or signaling networks necessary for viral entry. These primary receptors chiefly serve to temporally and spatially bring the viruses into close or direct contact with secondary receptors responsible for orchestrating virus internalization in a co-receptor system [1,5]. While these primary receptors may not functionally coordinate virus entry, they may additionally serve to activate downstream signaling cascades that play important roles in the propagation of infection.

Through inducing internalization, viruses are capable of causing an orchestration of cellular factors to further drive entry. Because of this, there are numerous pathways viruses can use to internalize into target cells including clathrin- and caveolin-mediated endocytosis, macropinocytosis, clathrin/caveolin-independent pathways, and also cholesterol dependent/independent routes [1]. Due to this stunning diversity in the ability of viruses to orchestrate internalization, virus entry is unsurprisingly complex to unravel.

### 3.1. Caveolin-/Lipid Raft-Mediated Endocytosis

**Figure 2:**
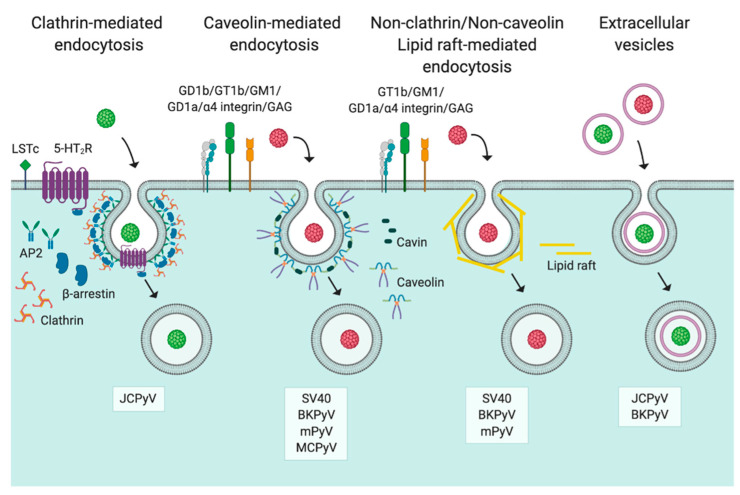
Entry pathways usurped by polyomaviruses. Polyomaviruses have been identified to hijack several of the currently known pathways to enter cells. JC polyomavirus (JCPyV) (green virion) is the only characterized polyomavirus to date that utilizes clathrin-mediated endocytosis, while SV40, BK polyomavirus (BKPyV), murine polyomavirus (mPyV), and Merkel cell polyomavirus (MCPyV) (red virion, collectively) usurp caveolin-mediated endocytosis and/or non-clathrin/non-caveolin lipid raft uptake mechanisms. JCPyV and BKPyV have also been affiliated with extracellular vesicles as a tactic for entry into target cells likely bypassing cellular attachment and entry factors. Figure created with BioRender.com.

Most polyomaviruses have been identified, at least in part, to use caveolin-mediated endocytosis for entry, except for JCPyV [80,81,82,83,84,85]. Characterized by the recruitment of caveolin, caveolin-mediated endocytosis involves a tight network of caveolins, primarily caveolin-1, an integral membrane protein. Upon recruitment, caveolins are capable of dimerization, which can be seen by electron microscopy [86] and is maintained by the actin cytoskeleton [87]. Caveolins serve to assist in the formation and stabilization of caveolae, flask-shaped invaginations at the cell surface. These invaginations consisting of lipid-rafts rich in caveolin (Figure 2), can also be found in the *trans*-Golgi network (TGN), and among specialized intracellular vesicles [16,88,89]. Notably, upon changes in membrane cholesterol, caveolins may localize to endosomes, the Golgi, or the endoplasmic reticulum (ER) [90,91], most of which are localization sites of polyomaviruses after entry [16,20,21,92,93,94,95].

SV40 was one of the first viruses identified to use caveolin-mediated endocytosis as a cellular entry mechanism [59,80,96]. Since this discovery, caveolin-mediated internalization has been demonstrated for other polyomaviruses including BKPyV, mPyV, and MCPyV, despite the usage of different receptors [21,81,94,97,98,99]. SV40 engages receptors GM1/MHC I and is then directed into clusters of caveolin, forming small, tight, flask-shaped invaginations [59]. The membrane invaginations are regulated by cavins, intracellular proteins that maintain caveolae morphology and dynamics through induction of membrane tubules [100]. After the recruitment of caveolin, dynamin II is recruited, acting as molecular scissors to pinch off the invagination through inherent GTPase function [92,101,102]. Following this, SV40 is sorted into early endosomes [103]. While SV40 was originally thought to be sorted into a “caveosome,” the existence of such a vesicle has since been deemed to be an artifact of overexpression of caveolin-1 protein [104]. Following this, investigation of SV40 association with vesicles following endocytosis was revisited. It was determined that SV40 internalization by caveolin-mediated endocytosis results in deposition of the virions into early endosomes followed by association with late endosomes [103]. Interestingly, the late endosomes also contained caveolin-1, and, upon overexpression, caveolin-1 also accumulated in endolysosomes, which was deemed to likely be an artifact, which had been previously associated with overexpression of caveolin-1 [103,104].

SV40 internalization into caveolae triggers actin breakdown and recruitment, suggesting the involvement of receptor tyrosine kinases in SV40 entry [21,102]. Receptor tyrosine kinases are often affiliated with ligand-mediated internalization of viruses by caveolin-/lipid-raft mediated endocytosis [2,105]. While clathrin-mediated endocytosis was initially thought to occur independently of tyrosine kinase activity, overlap between these two signaling pathways is becoming more appreciated, the importance of receptor tyrosine kinase signaling has been demonstrated for JCPyV infection [106]. Surprisingly, SV40 has also been demonstrated to enter into cells devoid of caveolin-1. In cells from a caveolin-1 knockout mouse SV40 internalization occurred more rapidly and is transported into pH neutral organelles [83].

Both caveolin-dependent and -independent internalization mechanisms have been identified for mPyV [81,107]. mPyV is deposited into early endosomes after entry rather than caveolin-1+ vesicles [108,109]. Like mPyV, BKPyV endocytosis also relies on interplay between the caveolar and endosomal endocytic systems [94,97]. In contrast, in renal proximal tubule endothelial cells, endocytosis of BKPyV occurs independently of clathrin or caveolin [99]. Together this highlights the importance of investigation of cell type-specific differences during viral infection.

Similar to SV40, MCPyV entry was found to depend on intracellular cholesterol, which is critical for caveolar- and lipid-raft mediated endocytosis, and cellular phosphatases including protein phosphatase 1 (PP1), 2A (PP2A), and 2B (PP2B) [21]. Furthermore, MCPyV internalization is dependent on actin dynamics as well as cellular GTPases, and Rho-like GTPase function [21]. The role of Rho-like GTPase in MCPyV entry is thought to occur through mediating actin-dependent endocytosis of MCPyV. Furthermore, MCPyV entry was not perturbed upon inhibition of clathrin or through inhibition of macropinocytosis via the use of EIPA [21].

### 3.2. Clathrin-Mediated Endocytosis

Unlike other polyomaviruses studied, JCPyV internalization has been identified to occur through clathrin-mediated endocytosis [106,110,111]. While this distinction occurs, it is unclear why JCPyV engagement of cellular receptors results in internalization by clathrin-mediated endocytosis (CME) in contrast to other polyomaviruses studied. CME serves as the most common entry pathway usurped by both enveloped and nonenveloped viruses [82,112]. CME consists of the interplay of numerous cellular factors including, but not limited to, clathrin, AP2, and dynamin, predominantly isoforms I and II [113,114,115,116,117,118,119,120]. Through sucrose-rich flotation assays, JCPyV internalization has been demonstrated to rely on intracellular cholesterol, as well as actin [121,122]. However, JCPyV entry is sensitive to chlorpromazine, an inhibitor of CME that also functions as a selective 5-hydroxytrypatime receptor (5-HTR) antagonist [110,123,124]. Importantly, this finding led to the hypothesis that JCPyV usurps the proteinaceous serotonin receptor family, subtype 2 (5-HT_2_R) for internalization, which was verified with inhibitors and antibodies to 5-HT_2_Rs as well as assays that specifically measure viral entry [111,123,125]. The 5-HT_2_Rs serve as entry receptors for JCPyV as overexpression of 5-HT_2_Rs does not enhance JCPyV binding [111,123], but specifically enhances viral entry [123]. In addition, sites of JCPyV infection are consistent with expression patterns of 5-HT_2_Rs, including expression in kidney and glial cells [126,127,128]. In further support, the recent literature has indicated an interaction between 5-HT_2_Rs and JCPyV through the utilization of a PLA assay [129]. In this report interactions were found to occur as early as 5 min postinfection but were transient, dissipating at 15 min postinternalization [129]. It is thought that the difference in internalization strategy usurped by JCPyV in comparison to other polyomaviruses is due to JCPyV requiring the additional proteinaceous cellular receptor for mediating viral internalization, 5-HT_2_Rs, though this remains unconfirmed though experimentation.

The determination that JCPyV likely uses CME for internalization was further supported by the finding that JCPyV entry was sensitive to siRNA targeting the heavy chain of clathrin, and JCPyV localizes with clathrin at time points consistent with viral internalization [111]. Interestingly, JCPyV internalization also relies on the endocytic protein β-arrestin, the first association of this protein with the promotion of virus entry [111,129]. β-arrestin binding domains within the 5-HT_2_R family, the Ala-Ser-Lys (ASK) motif as well as a proline, separated by six residues from a Asp-Arg-Tyr (DRY) motif on the third intracellular loop of 5-HT_2_Rs [130,131,132,133], have been demonstrated to be important for JCPyV internalization and infection [111,129]. After recruitment and formation of a clathrin-coated pit, JCPyV is delivered to an early endosome for intracellular trafficking dependent on the GTPase activity of dynamin I [111,114,134].

### 3.3. Non-Clathrin/Non-Caveolin Endocytosis

In recent years alternative endocytic pathways aside from the classic CME and caveolin-mediated endocytosis have also been described [135]. This pathway, labelled non-clathrin, non-caveolin endocytosis, has been demonstrated to rely on tyrosine-kinase activity and cholesterol while, in contrast to clathrin and caveolin uptake routes, being independent of dynamin [83]. One of the first published studies that began to describe this endocytic pathway was defined using SV40 in caveolin-1-devoid mouse embryonic fibroblast (MEFs) cells [83]. Upon uptake into these cells the viruses do not traffic by previously identified endocytic routes, and instead are delivered to the ER via cytosolic organelles that were nonendosomal in origin [83]. It is important to note that MEFs are not a commonly utilized cell model for studies of SV40 infection, which infects kidney cells of monkeys and humans [136]. Rather MEFs are utilized as an abortive model of infection, largely for studies of T-Ag transformation [137], and thus SV40 entry into these cells may differ from fully permissive cells.

It has been proposed that the non-clathrin non-caveolin uptake pathway is reliant on flotillins 1 and 2 [138,139]. Assembly of these two proteins promotes the formation of microdomains that bud into the cell [140]. Furthermore, GPI-linked proteins have been described to internalize into both flotillin-positive and caveolin-1+ vesicles suggesting that there may be overlap between these pathways and their induction by cellular receptors and/or ligands [140]. The activation of these two signaling pathways has since been linked to Src-family kinases and tyrosine-kinase activity, though much of their activation and crosstalk remains elusive [135,140,141,142]. Furthermore, the implication of this alternative route in uptake of polyomaviruses, aside from SV40, and its implications in viral pathogenesis remain to be elucidated.

### 3.4. Extracellular Vesicles

Recently, viruses have been identified to use extracellular vesicles (EVs) to infect cells [143]. EVs are cell membrane-derived vesicular structures that mediate transfer of material between cells [144]. Thus, as extracellular vehicles for transmitting material and mediating cellular communication, EVs can also transport viruses within the host. EVs have been associated with transporting both enveloped and nonenveloped viruses, including poliovirus, rhinovirus, hepatitis A and C viruses, coxsackievirus, dengue virus, rotavirus, and noroviruses [143]. These viruses have been identified to use EVs to transport either whole infectious virions or naked infectious genomes [143]. In transmitting virions, EVs can serve to enhance virulence of these viruses in multiple ways. EVs can increase the multiplicity of infection (numerous particles can be transmitted within a single EV), and EVs can protect naked infectious genomes and particles from neutralizing antibodies and from eliciting an immune response [143,145,146]. Furthermore, within EVs, viruses are protected from harsh chemicals or environments that would otherwise serve to neutralize infection [143]. EVs may be the answer to key questions that remain in transmission of viruses within a host between target cells or, on a larger scale, sites of infection. While many RNA viruses have been associated with the transport within EVs, more recently, DNA viruses, including polyomaviruses, have also been identified to utilize EVs [147,148,149]. Notably, EVs may serve as enticing modes of transmission for viruses as a means of infecting cells that do not express the typically required cellular attachment and/or entry factors [150].

BKPyV has been associated with the use of EVs for transmission within an infected host. These EVs, isolated from infected Vero and renal proximal tubular epithelial cells, contained tens of BKPyV particles and were capable of infecting cells independent of sialylated glycan expression, suggesting that an alternative entry pathway is used to establish infection [149]. The identification that BKPyV may use EVs may indicate mechanisms of persistence and immune evasion of this virus in the asymptomatically infected host, prior to the onset of virus-associated disease.

A recent publication identified that JC polyomavirus-infected glial SVG-A cells released EVs into the medium. These EVs, approximately 100–200 nm in size, contained infectious virions, and appeared to be derived from either multivesicular bodies or the plasma membrane. Importantly, JCPyV-containing EVs were capable of eliciting an infection in naïve cells independent of attachment factor LSTc and entry 5-HT_2_ receptors [147]. This mode of transport of JCPyV is proposed as a means for transmission of the virus from sites of persistent infection, the kidney, to the central nervous system (CNS), for the development of PML. This is further supported by findings that oligodendrocytes and astrocytes, cell types targeted by JCPyV in the CNS [151], have been reported to lack expression of attachment factor LSTc, through the analysis of tissue sections [128], and that strains of JCPyV isolated from PML patient populations contain mutations within the LSTc binding site of VP1 [152]. However, incubation of JCPyV with LSTc pentasaccharide blocks attachment and infection in SVG-A cells, a mixed population of immortalized astrocytes and oligodendrocytes [74]. Together this emphasizes the importance of using various types of relevant cell models to study viral infection and highlights that there may be cell-type dependent differences and multiple entry strategies utilized simultaneously. Moreover, archetype JCPyV DNA was found in extracellular vesicles of human-derived plasma from both healthy and HIV+ individuals, those at significant risk for development of PML [6,148]. The potential significance of these findings for JCPyV virulence and tropism cannot be understated.

## 4. Trafficking

**Figure 3:**
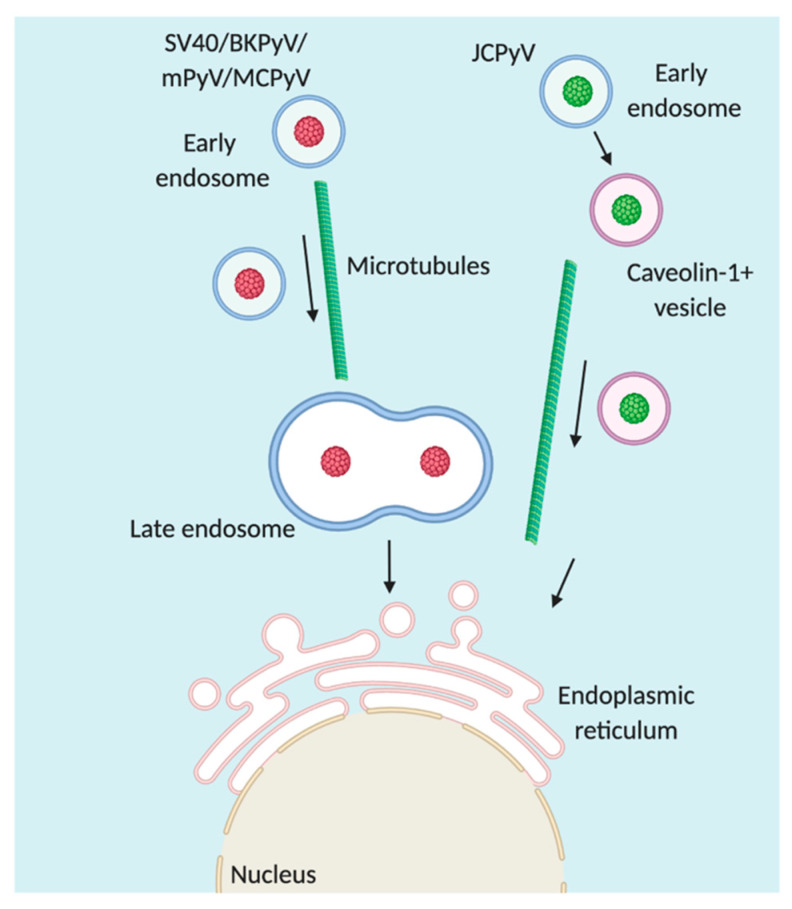
Intracellular trafficking of polyomaviruses. After internalization of polyomaviruses into the cell, they are targeted to the endoplasmic reticulum (ER). Upon internalization of SV40, BKPyV, mPyV, or MCPyV (red virion, collectively) into a pH neutral early endosome, the virus is delivered to the ER via microtubule-mediated transport and the late endosome/lysosome. Meanwhile, upon internalization of JCPyV (green virion) into an early endosome, association with caveolin-1+ vesicles occurs prior to retrograde transport to the ER. Figure created with BioRender.com.

One theme is apparent among polyomaviruses; while these viruses are attributed to using distinct cellular attachment and entry factors and enter cells by differing mechanisms, all polyomaviruses are targeted to the ER [15,16,17,18,19,20,21]. It is postulated that direct interactions among polyomaviruses and their respective receptors largely dictate intracellular trafficking [5]. Regardless, targeting to the ER is critical for progression of the viral infectious cycle as disruption of ER trafficking halts the progression of infection [16,20,21]. To reach the ER, polyomaviruses have been attributed to following various trafficking routes (depicted in Figure 3).

Typical progression of the endosomal–lysosomal system begins at the early endosome. The early endosome is marked by the intracellular protein Rab5 [153]. Rab proteins serve to control the trafficking, fusion, sorting, and maturation of endosomes [153]. The early endosome serves as the first major sorting hub and from this hub, cargo proteins can be sent to recycling Rab11+ endosomes for recycling to the cell surface, by retrograde transport to late Rab7+ endosomes for targeting to the trans-Golgi network (TGN) or the ER, or to the lysosome for degradation [154]. After internalization, JCPyV colocalizes with Rab5+ endosomes as early as 15 min post infection [122]. Localization of JCPyV to early endosomes is critical for the progression of infection; expression of dominant-negative mutants of Rab5 inhibits infectivity [122]. Interestingly, after localization to early endosomes, JCPyV appears to exit the endosomal system and is transferred to a caveolin-1+ vesicle [122]. How JCPyV leaves the endosomal/lysosomal system remains unclear. Treatment of cells with either inhibitors or dominant negative constructs directed towards recycling (Rab11) or late (Rab7) endosomes does not impair infection [20,121,122]. Furthermore, Retro-2, a compound that prevents ER transport from the endosome by an undefined mechanism, hinders JCPyV-ER localization [95], and cellular depletion of caveolin-1 or sequestration of intracellular cholesterol reduces JCPyV infection [122]. While originally thought to be distinct intracellular pathways, crossover between the endosomal and caveolar systems is becoming more appreciated [155,156]; although JCPyV is internalized by CME, it still requires caveolin for transport to the ER, like other polyomaviruses studied [20].

Following entry of mPyV by caveolin-dependent/-independent endocytosis, mPyV is also deposited into an early endosome [108]. From the early endosome, mPyV is transported into a late endosome via microtubule-dependent transport [108]. Within the late endosome, low pH (5.5) induces conformational capsid changes that allow for enhanced disassembly within the ER [109]. The use of agents that prevent the pH drop in the endosome within these systems inhibits mPyV infection [108,109]. From the endolysosomal system, mPyV is sorted to the ER for further disassembly [56,109].

Similar to mPyV, BKPyV has also been attributed to require low pH prior to localization within the ER [97,157]. Recently, the anti-diabetic drug, glibenclamide, which blocks the cystic fibrosis transmembrane conductance regulator (CFTR), impeded BKPyV infection [158]. Importantly, the maximum inhibitory effect of this drug was found to be 4 hpi, suggesting that the hindrance in infection occurs during trafficking of the virus to the ER [158]. Collectively this highlights the importance of ion channel regulation during endosomal trafficking of BKPyV.

After sorting into a caveolin-1+ late endosome, SV40 is transported by microtubules to the ER [16]. Trafficking of SV40 through the caveolar/endosomal systems is reliant on acidification of the endosome as well as ionic balance of Ca^2+^ [159]. Unsurprisingly, trafficking of MCPyV also involves the caveolar and endosomal systems, also relying on regulation of endosomal Ca^2+^; however, transport of MCPyV also depends on the balance of K^+^, of which is dispensable for SV40 infection [159]. This suggests that intracellular transport of polyomaviruses must be tightly regulated for infection to be successful, while also providing the appropriate tailored environment for individual polyomaviruses. After arrival into an early endosome by caveolin-mediated endocytosis, MCPyV traffics to late endosomes via microtubules. Interestingly, within the endosomal compartment, MCPyV has been described to acquire a lipid envelope [21]. This envelope is wrapped tightly around the virion and is posited to become acquired within the late endosomal system rather than in specialized compartments. The authors speculate that this envelope may be a new antiviral defense mechanism or a means of evading the low pH of the maturing/late endosomal compartments prior to arrival in the ER [21].

How polyomaviruses reach the ER from the endosomal/lysosomal system largely remains enigmatic. For mPyV, direct binding between mPyV and ganglioside GD1a results in targeting of mPyV from the late endosome to the ER [107]. After sorting into late endosomes, BKPyV colocalizes with Rab18, syntaxin 18, a member of the ER membrane fusion system, and NRZ, which form a complex. This complex serves to anchor BKPyV-containing vesicles at the ER. In the absence of Rab18, BKPyV becomes trapped in the late endosome and ER transport does not occur [160]. Given the conservation among polyomavirus transport pathways, whether Rab18/syntaxin 18/NRZ complexes play a role in late sorting of other polyomaviruses to the ER warrants exploration.

## 5. Disassembly within the Endoplasmic Reticulum

**Figure 4:**
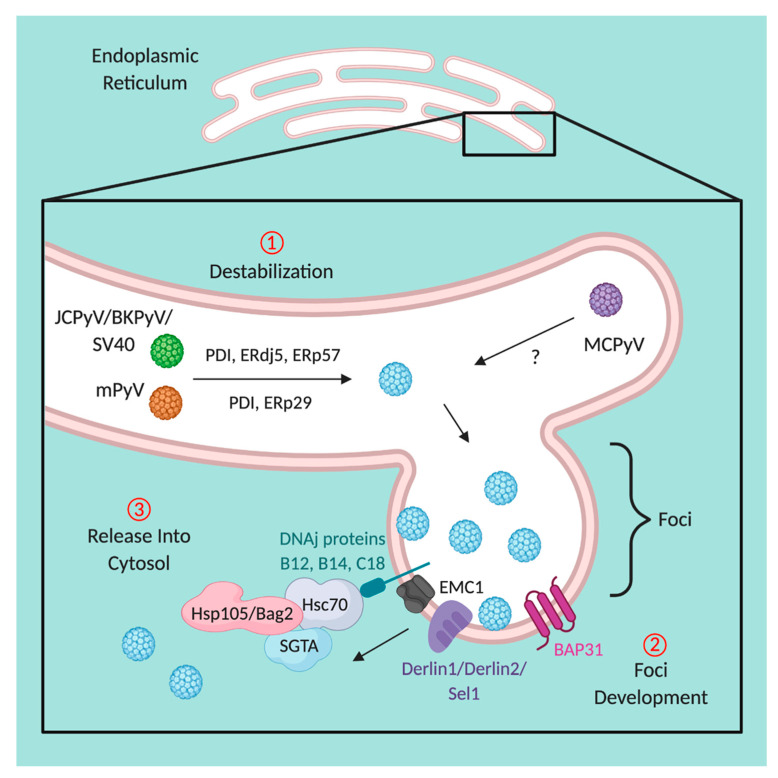
Release of polyomaviruses from the ER to the cytosol. (1) Upon arrival of polyomaviruses JCPyV, BKPyV, SV40 (green virion, collectively), mPyV (orange virion), and MCPyV (purple virion) in the ER, the viral capsids are destabilized due to reduction and isomerization of disulfide bonds by ER redox proteins, PDI, ERdj5, ERp57, or ERp29. Destabilization of MCPyV remains elusive (indicated by ‘?’). (2) The now-hydrophobic particle embeds itself in the ER membrane and is stabilized by EMC1, preventing further disassembly of the viral capsid. As demonstrated by SV40 (potential pathway of PyV convergence indicated by blue virion), localization of the virus in the membrane of the ER induces the relocation of DNA J proteins (B12, B14, C18) and BAP31 to the site, forming a foci. (3) Direct interactions between DNA J proteins and cytosolic extraction machinery (Hsc70, HSP105, Bag2, and SGTA) form a complex, and with the assistance of derlin1, derlin2, or sel1, facilitate the extraction of the virus across the ER membrane and into the cytosol, primed for arrival at the nucleus. Figure created with BioRender.com.

While it has been established for some time that polyomaviruses require transit through the ER for completion of the infectious cycle [16,20,21], discoveries made in recent years have just begun to unravel this complex process. Much of what is known about disassembly within the ER is largely due to advances for SV40. The capsid of polyomaviruses is stabilized by covalent bonds, which upon arrival in the ER, are reduced and isomerized by ER-resident redox proteins PDI, ERdj5, and ERp57 (Figure 4, step 1) [161]. Furthermore, mPyV also requires a redox environment for capsid destabilization; though differing from SV40, ERp57 is dispensable for infection, instead relying on ERp29 for disassembly [162,163,164]. In this process, ERp29 acts as a chaperone to unravel the C-terminal linkages of VP1, the interactions that stabilize the overall architecture of the capsid; because of this, the hydrophobic capsid proteins, VP2 and VP3, become exposed [162,164]. Similar to mPyV, JCPyV also usurps ERp29 for isomerization, as siRNA targeting ERp29, ERp57, and ERp72 all resulted in diminished infectivity [20]. It is important to note that ERp29 is not capable of disrupting the disulfide bonds of the viral capsid as it contains only one cysteine residue, highlighting the importance of isomerization for the progression of viral infection [165]. While MCPyV has recently been demonstrated to traffic to the ER, similarly to other polyomaviruses [21], whether or not destabilization by ER-resident redox proteins is necessary for infection has yet to be determined.

As the disulfide bonds in the capsid of polyomaviruses are reduced and isomerized, the now-hydrophobic virion embeds itself in the membrane of the ER through the N-terminus of the exposed VP2, mimicking a misfolded protein [166]. In doing so, SV40 has been demonstrated to cause the formation of a foci, or penetration site, in the ER membrane (Figure 4, step 2) [19,166,167,168,169]. Virions devoid of VP2 arrive at the ER but are incapable of release from the ER and the progression of infection [166]. The formation of foci in the ER leads to the recruitment of membrane-bound proteins to the site of penetration, including BAP31 and BiP, typically affiliated with ER-associated degradation (ERAD) of misfolded proteins [166]. Within the membrane, direct interactions between the membrane chaperone EMC1 and the virus occur, preventing further premature disassembly of the virion while in the ER [170]. Additional membrane-bound proteins are recruited to the foci, including DNA J proteins B12, B14, and C18 as well as cytosolic chaperones Hsc70, Hsc105, Bag2, and SGTA that function to extract SV40 through the ER membrane and into the cytosol (Figure 4, step 3) [167,168,171,172,173]. This extraction is further chaperoned by membrane proteins derlin1 and sel1 (SV40/BKPyV/JCPyV) or derlin2 (mPyV), or in the case of JCPyV, potentially through sel1, independent of derlin proteins [20,93,157,161].

Importantly, the transmission of SV40 from the ER into the cytosol appears to be tightly regulated. Numerous publications highlight the critical nature of the foci for delivery of SV40 into the cytosol: (1) if the foci are not formed properly, infection does not progress [167,169,170], (2) the foci are formed prior to cytosolic delivery of the virions [167,169,170,172,173], (3) hydrophobic virions cluster to foci prior to release [19,169,174], and (4) disruption of cytosolic proteins that mediate extraction results in trapped virions within the ER [167,172,173]. It has been recently demonstrated that the formation of foci by SV40 involves the recruitment of cytosolic kinesin-1 to form the structure budding from the ER [174], though how SV40 recruits kinesin-1 while still residing within the ER is unclear. Once SV40 has been extracted from the ER into the cytosol, mechanical stress exerted by dynein-1 results in the further disassembly of the viral particle, priming the virus for nuclear localization [175], similar to that described for kinesin-1-mediated disassembly of adenovirus [176]. Moreover, the impact of Hsc70 cannot be discounted, as Hsc70-mediated cytosolic disassembly of polyomavirus capsids has been described for SV40 [167,168,174,175] and for mPyV [177]. In fact, interactions between hydrophobic polyomavirus capsid proteins and cytosolic chaperones may play a significant role in preventing the targeted degradation of the viruses, subverting the canonical fate of misfolded proteins [167,178].

Until recently, a major question remained, how does a particle the size of a polyomavirus move through the ER membrane without disrupting the overall functionality of the organelle? Movement of large macromolecules likely induces significant mechanical stress on the ER, which may result in reduced structural integrity [179]. In moving through the membrane, SV40 has been demonstrated to co-opt ER-membrane-resident reticulon (RTN), responsible for maintaining the overall morphology of the ER membrane [179]. Under normal function, RTN maintains the structure of the ER membrane during movement of misfolded proteins from the ER into the cytosol, typically targeted for degradation. RTN aids in movement of SV40 by assisting in membrane curvature and flexibility during release [179], allowing for the membrane to undergo significantly more mechanical stress than what is normally tolerated.

Polyomavirus exploitation of the ERAD pathway is critical for partial disassembly of the viral capsid, highlighting the importance of ER arrival for all polyomaviruses studied to date. Partial disassembly of the viral capsid is essential for nuclear transport of polyomavirus virions as disassembly releases the internal genetic material. As dsDNA viruses, arrival in the nucleus is critical for transcription and replication. Disruption of any proteins utilized by these viruses between the plasma membrane and arrival of the viruses at the nucleus prevents transcription and thus replication, highlighting the vital regulation of these processes and their importance for the propagation of viral infection.

## 6. Nuclear Transport of Polyomaviruses

In contrast to small molecules (smaller than 9 nm), large macromolecules cannot transit through the nuclear pore complex (NPC) by passive diffusion. Transport of larger molecules across the nuclear membrane (approximately 45 nm) occurs via karyopherin-β family proteins comprising importin transport factors for transit into the nucleus, and exportins for transit out of the nucleus [180]. Importantly, importins α and β heterodimerize, and this dimerization is critical for transport of the cargo to the nucleus [181]. However, importin β has also been associated with mediating transport out of the nucleus [180,181]. Association of importins with cargo occurs in the cytoplasm where they then act as chaperones, facilitating the movement of the cargo across the nuclear membrane, through first docking with nucleoporins and then releasing the cargo on the opposite side of the membrane via GTPase hydrolysis [180]. Usurpation of these importins to guide nuclear transport of viruses have been well described [182]. In order to hijack the host’s nuclear transport machinery, a nuclear localization sequence (NLS) must become exposed, thereby acting as a ticket or voucher for nuclear transport. Importin β recognizes and binds to the NLS of the cargo in the cytoplasm while importin β directly interacts with the nucleoporins [183].

Exposure of polyomavirus NLS sequences occurs during uncoating within the ER. Direct interactions between the NLS of SV40s VP2 and VP3 with the importin complex are necessary for arrival of SV40 within the nucleus [184,185]. While VP1 of SV40 also contains an NLS, interactions between this NLS and importins are not critical for infection [184]. In addition to SV40, disruption of the NLS of BKPyV VP2 and VP3 significantly impairs infection [186]. However, in contrast to BKPyV and SV40, interaction between the NLS of JCPyV VP1 and the importin complex is sufficient to support JCPyV infection, as demonstrated by virus-like particles (VLPs) comprised only of DNA and VP1 [187]. For mPyV, it was recently determined that the NLS of VP1 is sufficient to allow for nuclear transport, though the NLSs of VP2/3 may also contribute in a smaller capacity [188]. Interestingly, the authors noted that only a subset of the viral protein associated with importins is delivered into the nucleus [188]; this is in agreement with a separate report that SV40 association with importins α/β was an infrequently occurring event [185]. Several possibilities to explain this occurrence were rationalized by Soldatova et al.: (1) over the course of infection only a subset of virions arrive in the cytoplasm for transport to the nucleus during entry, (2) upon arrival of the partially disassembled particles into the cytoplasm, a subset of these particles are likely targeted for degradation, and (3) transport of the particles into the nucleus must overcome barriers including detection by the innate immune system [188]. However, direct transport of viral particles may occur through the leaflets of the ER directly into the nucleus. Though not well characterized, it has been suggested that infection by SV40 may result in breakdown of the nuclear envelope, allowing for direct ER to nucleus transport [189]. This is conflicting with additional reports that SV40 requires transit through the cytoplasm prior to nuclear arrival for infection, identified through the implementation of anti-VP1 and -VP3 antibodies injected into the cytoplasm resulting in blocked infection [190]. This is further supported by the characterization of multiple cytosolic proteins in productive SV40 infection [167,168,171,172,173]. However, the implication of destabilization of the nuclear membrane during the promotion of viral infection cannot be discounted, as this may be necessary to allow for increased nuclear localization of SV40 [189,191]. Aside from these data, there is limited understanding of the complex interactions that underscore the arrival of polyomavirus genetic material within the nucleus, highlighting a critical gap in our understanding of this event in the promotion of virus entry and thus infection.

## 7. Concluding Remarks

In the last few decades, numerous advances have been made to better define the infectious cycle of polyomaviruses from start to finish. Understanding the complex interactions that occur between a viral pathogen and target host cells are key to the development of improved therapies for the treatment of virus-associated diseases. Of the polyomaviruses discussed in this review, four have been associated with human illness, BKPyV, JCPyV, TSPyV, and MCPyV. In usurping specific host cellular receptors and hijacking signaling networks, viruses co-opt intracellular endocytic and signaling pathways that orchestrate delivery of the virus to the appropriate cellular compartment for replication. Delivery of the virus to the right compartment, at the right time, is tightly regulated, ensuring replicative success; failing to do so results in the disruption of viral infection either through nonproductive infection routes or through intracellular detection of the virus by the host immune system. Thus, in order to cause infection, polyomaviruses must either activate host cellular receptors and hijack signaling networks or usurp EVs to mediate their internalization within the cell.

Although much of the literature highlighted in this work underscores the importance of the usurpation of specific cellular receptors and their necessity for dictating downstream signaling events, EVs appear to contradict this evidence. How viral utilization of EVs results in the activation of signaling networks that are necessary for the promotion of viral infection, while bypassing the activation of cellular receptors, remains unclear. However, the likelihood is that polyomaviruses hijack EVs for maintaining viral persistence within the host and for immune system avoidance. In releasing infectious particles packaged into EVs, polyomaviruses may avoid cellular lysis, which may trigger clearance of the infection [192]. This is supported by evidence that there is low spread of BKPyV particles from infected cells to naive cells during persistent infection [193]. However, the mechanisms by which polyomaviruses may utilize EVs to target particular tissues or organs, previously thought not to be susceptible, is not clear. Moreover, utilizing this method may result in increased spread of viral infection and heightened development of polyomavirus-associated disease [192]. Further investigation into the contribution of EVs to the spread of polyomavirus infection and their implications for facilitating the necessary signaling networks within cells in order to promote infection is warranted.

For many years it was not well-understood why polyomaviruses target the ER upon internalization inside the cell, by far the most unique feature of polyomavirus infection. Through significant advances we now better understand the importance of the ER in viral disassembly for polyomavirus infection. However, many key questions remain to be addressed. It remains unclear why polyomaviruses target a broad diversity of cellular attachment and entry factors to eventually arrive at the same destination, especially when the overall conformation of the cellular attachment protein, VP1, is highly conserved. Furthermore, how polyomaviruses direct their intracellular movement to the ER upon internalization remains largely uncharacterized, including transit from intracellular vesicles into the ER itself. In addition, it is important to highlight that much of our understanding of polyomavirus usurpation of the ER relies on what has been identified for SV40. While disassembly machinery and mechanisms utilized by polyomaviruses in the ER may demonstrate some conservation, it remains possible that there are unique factors and pathways utilized, as is the case for PyV entry. While the necessity of the ER for other non-human polyomaviruses and their human counterparts has been determined, differences have already emerged in the involvement of specific components of the ERAD machinery. Clearly, there is still much to learn about the redox environment of the ER for specific polyomaviruses and the impact this plays for progression of infection.

While significant progress has been made to unravel the complexities of polyomavirus infection multiple roadblocks still exist in our understanding of these multifaceted processes. The implication of cell-type specific differences as well as the utilization of multiple receptors and endocytic pathways during infection further complicate our understanding of polyomavirus infection. Furthermore, the utilization of virus-like particles (VLPs) in comparison to whole virions and their potentially differing attachment, entry, and trafficking properties cannot be ignored as much of these potential differences remain elusive. These studies may be further complicated by the notion that viral infection could occur by receptor-mediated attachment and entry of virions and EV-mediated uptake simultaneously, thus complicating our understanding of these processes.

However, due to the high similarity among polyomaviruses, insights from the study of polyomavirus infection, from attachment to arrival at the nucleus, will undoubtedly advance our understanding of viral infection strategies as a whole. Knowledge gained from future studies in addition to those summarized in this work provide insight into the development of antiviral interventions for the treatment of human polyomavirus-associated disease.
